# Core–Shell Structure Trimetallic Sulfide@N-Doped Carbon Composites as Anodes for Enhanced Lithium-Ion Storage Performance

**DOI:** 10.3390/molecules28227580

**Published:** 2023-11-14

**Authors:** Xiuyan Li, Liangxing Zhu, Chenyu Yang, Yinan Wang, Shaonan Gu, Guowei Zhou

**Affiliations:** 1 School of Chemical Engineering and Environment, Shandong Peninsula Engineering Research Center of Comprehensive Brine Utilization, Weifang University of Science and Technology, Weifang 262700, China; iamlixiuyan@126.com; 2 Key Laboratory of Fine Chemicals in Universities of Shandong, Jinan Engineering Laboratory for Multi-Scale Functional Materials, School of Chemistry and Chemical Engineering, Qilu University of Technology (Shandong Academy of Sciences), Jinan 250353, China; zlx112411@163.com (L.Z.); yangchenyu199802@163.com (C.Y.); sngu@qlu.edu.cn (S.G.)

**Keywords:** core–shell structure, trimetallic sulfide, lithium-ion batteries, anode material

## Abstract

The high specific capacity of transition metal sulfides (TMSs) opens up a promising new development direction for lithium-ion batteries with high energy storage. However, the poor conductivity and serious volume expansion during charge and discharge hinder their further development. In this work, trimetallic sulfide Zn–Co–Fe–S@nitrogen-doped carbon (Zn–Co–Fe–S@N–C) polyhedron composite with a core–shell structure is synthesized through a simple self-template method using ZnCoFe–ZIF as precursor, followed by a dopamine surface polymerization process and sulfidation during high-temperature calcination. The obvious space between the internal core and the external shell of the Zn–Co–Fe–S@N–C composites can effectively alleviate the volume expansion and shorten the diffusion path of Li ions during charge and discharge cycles. The nitrogen-doped carbon shell not only significantly improves the electrical conductivity of the material, but also strengthens the structural stability of the material. The synergistic effect between polymetallic sulfides improves the electrochemical reactivity. When used as an anode in lithium-ion batteries (LIBs), the prepared Zn–Co–Fe–S@N–C composite exhibits a high specific capacity retention (966.6 mA h g^−1^ after 100 cycles at current rate of 100 mA g^−1^) and good cyclic stability (499.17 mA h g^−1^ after 120 cycles at current rate of 2000 mA g^−1^).

## 1. Introduction

Lithium-ion batteries (LIBs), as a new generation of rechargeable batteries, have been widely applied in the field of energy storage and conversion [1,2]. Compared to other rechargeable systems, LIBs deliver unrivaled high power and energy density as well as incredible cycling stability. The application of various portable electronic devices and new energy electric vehicles requires the development of high-performance LIBs [3,4]. The development of new electrode materials with new compositions or structures, especially anode materials, is one of the keys to enhancing battery performance. Therefore, various anode materials, such as metal oxides [5,6,7], phosphates [8,9] and sulfides [10,11,12], are widely studied as anode materials for LIBs. Transition metal sulfides (TMSs) have been widely used in the field of energy storage and conversion as anode materials because of their high theoretical capacity, strong oxidation–reduction capability, good safety and low cost [13,14]. For instance, Nong et al. synthesized bimetallic sulfide carbon microrods (Co_9_S_8_/ZnS@C MRs) consisting of a Co_9_S_8_/ZnS core and outer carbon shells, showing excellent cyclic stability [15]. Jin et al. synthesized NiS_1.03_@NiMoS_4_@C composites by encapsulating NiS_1.03_@NiMoS_4_ nanocrystals in mesoporous carbon microspheres, which showed high reversible capacity and good cyclic stability [16]. However, due to the large volume expansion and poor conductivity during charge and discharge, the cyclic stability and rate performance are always poor, which seriously hinder its practical application in LIBs [17,18].

As a novel kind of ordered crystalline materials, one of the biggest advantages of metal–organic frameworks (MOFs) is that the composition is adjustable, the morphology is controllable and an MOF material can contain a variety of different metal ions/clusters and organic ligands [19]. This provides a variety of possibilities for the preparation of hollow structural materials, with their own inherently adjustable pore structures and geometric structures composed of metal centers and organic ligands, and a variety of unique hollow structures can be obtained through the controlled heat treatment of MOFs in various gas atmospheres [20]. If MOFs are heated in an inert gas atmosphere, the organic ligand will be carbonized into a carbon shell covering the outside of the metal or metal compound, which maintains the inherently porous properties of the MOFs, thereby improving structural integrity and increasing the electronic conductivity of the electrode materials. Therefore, MOFs are ideal precursors for the preparation of various hollow nanomaterials, which have great application potential in the field of energy storage [21]. Meanwhile, the introduction of carbon materials to prepare composites can be used as a conductive buffer medium to solve inherent problems, such as poor conductivity and large volume changes in the current electrode materials, in order to realize enhanced cyclic stability and coulombic efficiency; this is undoubtedly a promising and effective method for electrode materials for green and renewable energy storage devices.

However, defects such as the slow ion migration kinetics and poor structural stability of metal sulfides due to severe bulk effects as well as low intrinsic conductivity have led to poor multiplicative performance and rapid capacity decay of such electrode materials during cycling, which greatly hinder the development and practical application of metal sulfides [22]. In order to promote the application of TMSs in anode materials, a lot of efforts have been made and effective methods have been proposed. Among them, tuning the component of TMS composites is an effective method to improve their electrochemical performance. It was found that multiphase metal sulfides have the advantages of significant capacity enhancement and abundant redox potentials [23]. However, compositional modulation alone still cannot ensure the rapid diffusion of Li^+^ or structural stabilization. To address the current problems, researchers have made efforts to reduce ion diffusion distances and buffer structural strains, mainly by designing nanostructures of metal sulfides. Therefore, it is necessary to design hollow structures that could reasonably alleviate the volume expansion of TMSs, while improving electrical conductivity and Li^+^ ion diffusion [24,25,26]. Among them, due to the particularity of its structure, the core–shell structure can not only provide a larger void to alleviate volume expansion during the ion insertion/extraction process, but also has a higher porosity and a larger specific surface area, thereby accelerating the penetration of the electrolyte and increasing the contact area between the material and the electrolyte and improving the electrochemical performance [27]. Core–shell hollow nanostructures are a special class of nanomaterials with well-defined solid shells and internal voids that have attracted considerable interest due to their unique structural features. In energy-related applications, this novel core–shell structure simultaneously offers excellent electrical conductivity, fast ion transport kinetics and outstanding structural stability. Thanks to it having abundant active sites, fast ion mobility, strong electronic conductivity and good structural stability, the complex internal structure improves volumetric energy density by increasing the weight fraction of active sites and the power density and prolongs the cycle life due to enhanced structural stability [28]. Aslam et al. proposed a single-pore hollow core–shell ZnCoS@Co_9_S_8_/N-doped carbon composite material [29]. Due to its unique nanostructure and the synergistic effect of its components, it showed excellent reversible performance and good cycle stability when applied to LIBs. Wei et al. designed a nanostructured ternary TMS-coupled N/S-doped carbon protector using a strategy of composition regulation and structure protection (NiCoFeS@NSC) [30]. As an anode of LIBs, the electrode has a specific capacity of 995.7 mA h g^−1^ after 1000 cycles of 1 A g^−1^. Due to its complex structure and multicomponent composition, the synthesis of complex hollow micro/nanostructured metal oxide composites with multi-elemental compositions and novel morphologies can be achieved through various routes, but the development of simple and efficient synthesis methods is still very challenging [31]. Therefore, the rational design of hollow structural materials is an effective way to solve the problem of volume expansion in LIB anode materials.

Herein, we designed and synthesized core–shell trimetallic sulfide Zn–Co–Fe–S@nitrogen-doped carbon (Zn–Co–Fe–S@N–C) polyhedron composites. When applied as an anode material for LIBs, the specific capacity is 966.6 mA h g^−1^ after 100 cycles at a current density of 100 mA g^−1^. In particular, even at the high current density of 2000 mA g^−1^, the specific capacity of the trimetallic core–shell Zn–Co–Fe–S@N–C polyhedron can still reach 497.72 mA h g^−1^ after 120 cycles. The high-quality electrochemical performance is largely because the inimitable core–shell structure can provide sufficient void space to mitigate the volume expansion effects generated during the Li^+^ insertion/desertion reaction process and shorten the diffusion path of ions/electrons.

## 2. Results and Discussion

**Scheme 1:**
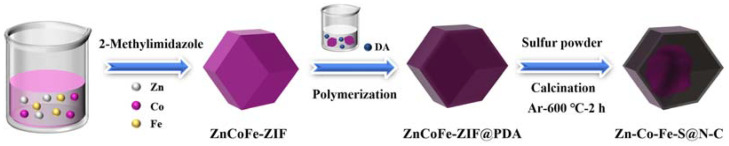
Diagram of the formation process of the Zn–Co–Fe–S@N–C core–shell polyhedron.

The formation mechanism of the core–shell structure Zn–Co–Fe–S@N–C polyhedron composites is shown in Scheme 1. First, 2-methylimidazole coordinated with Fe^2+^, Co^2+^ and Zn^2+^ ions to form the FeCoZn–Zeolitic Imidazolate Framework (ZIF) precursor. Then, the precursor was further coated with polydopamine (PDA) through the surface polymerization process and finally carbonized with sulfur powder under Ar at 600 °C. In the process of high-temperature calcination and vulcanization, Fe^2+^, Co^2+^ and Zn^2+^ ions in the ZnCoFe–ZIF framework were transferred to cobalt/zinc/iron metal sulfides. At the same time, the PDA shell was carbonized to a nitrogen-doped carbon shell. Finally, we successfully prepared the Zn–Co–Fe–S@N–C dodecahedron with a core–shell structure.

**Figure 1:**
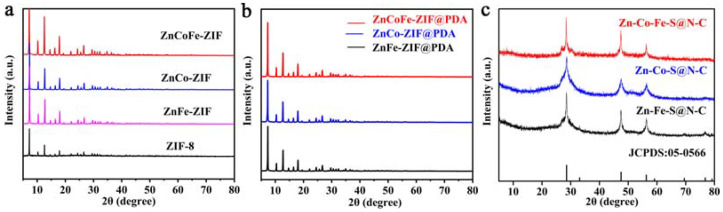
XRD patterns of (**a**) ZnCoFe–ZIF, ZnCo–ZIF and ZnFe–ZIF; (**b**) ZnCoFe–ZIF@PDA, ZnCo–ZIF@PDA and ZnFe–ZIF@PDA; (**c**) Zn–Co–S@N–C, Zn–Fe–S@N–C and Zn–Co–Fe–S@N–C.

The synthesized materials were characterized via XRD to determine their crystalline structures. Figure 1a shows the XRD patterns of the precursors ZnCoFe–ZIF, ZnCo–ZIF and ZnFe–ZIF. It can be seen from the figure that the diffraction peaks of the precursors ZnCoFe–ZIF, ZnCo–ZIF and ZnFe–ZIF are well matched with those of the previously synthesized ZIF–8, which confirms that the doping of Fe and Co did not induce the formation of Fe- and Co-based impurities. Figure 1b shows the XRD patterns of ZnCoFe–ZIF@PDA, ZnCo–ZIF@PDA and ZnFe–ZIF@PDA after coating with PDA. There is no significant difference between the diffraction peaks of the precursors, indicating that coating with PDA did not introduce impurities. Figure 1c shows the XRD patterns of Zn–Co–Fe–S@N–C, Zn–Co–S@N–C and Zn–Fe–S@N–C obtained after calcination with sulfur powder at a high temperature. The diffraction peaks of Zn–Co–Fe–S@N–C, Zn–Co–S@N–C and Zn–Fe–S@N–C at 28.5°, 47.5°, 56.2°, 69.5° and 76.8° correspond to those of the (111), (220), (311), (400) and (331) crystalline planes of the ZnS (JCPDS: 05-0566), which further explains that there were no impurities formed in the as-synthesized samples [32].

**Figure 2:**
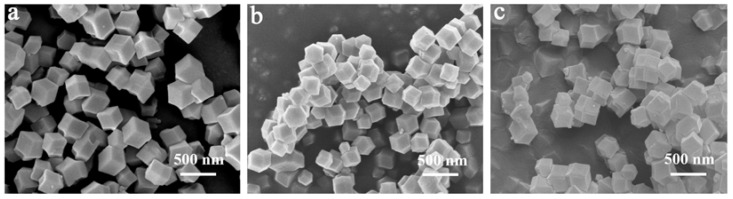
SEM images of precursors (**a**) ZnCoFe–ZIF, (**b**) ZnFe–ZIF and (**c**) ZnCo–ZIF.

**Figure 3:**
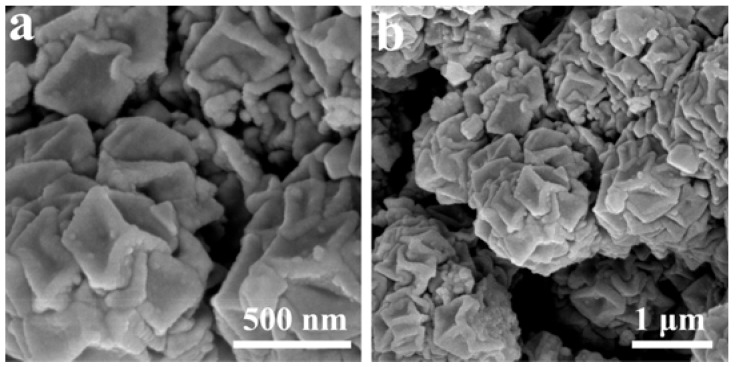
(**a**,**b**) SEM images of Zn–Co–Fe–S@N–C at different magnification.

The morphology of the precursors and products were observed using FESEM. Figure 2a shows the SEM image of the precursor ZnCoFe–ZIF, Figure 2b shows the SEM image of the precursor ZnFe–ZIF, and Figure 2c shows the SEM image of the precursor ZnCo–ZIF. It can be seen from the figures that the precursors are all polyhedrons with smooth surfaces, uniform size and the same shape, indicating the successful doping of Co and Fe instead of the growth of an impurity compound on the surface. During calcination, the surface of the polyhedrons became rough and wrinkled, but still maintained the morphology as shown in Figure 3a,b.

**Figure 4:**
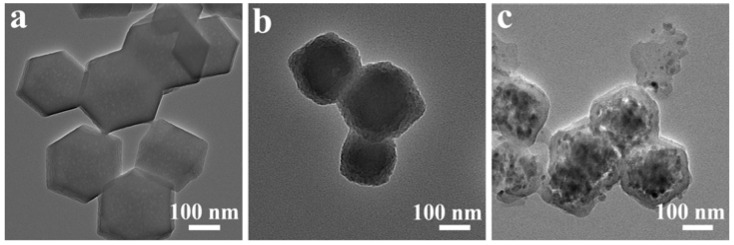
TEM images of (**a**) ZnCoFe–ZIF; (**b**) ZnCoFe–ZIF@PDA; (**c**) Zn–Co–Fe–S@N–C.

In order to better observe the internal structure of the material, TEM analysis was performed, as shown in Figure 4. Figure 4a is the TEM image of the precursor ZnCoFe–ZIF, which shows that it has a uniform solid structure inside and a relatively smooth surface. Figure 4b is the TEM image of ZnCoFe–ZIF after coating with PDA, which shows that the surface of ZnCoFe–ZIF@PDA is relatively rough, indicating the successful coating of dopamine. The TEM image of Zn–Co–Fe–S@N–C after calcination is shown in Figure 4c, from which a clear shell and inner core can be observed, and the core–shell structure has been successfully prepared. The core–shell structure, by virtue of its unique structural advantages, has a higher weight fraction of reactive material, complex internal voids and a larger specific surface area, which would be favorable for improving electrical conductivity and accelerating ion transport kinetics.

**Figure 5:**
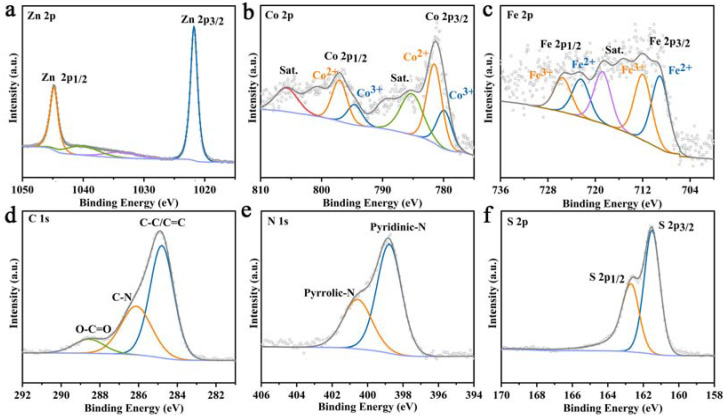
XPS energy spectra of Zn–Co–Fe–S@N–C: (**a**) Zn 2p; (**b**) Co 2p; (**c**) Fe 2p; (**d**) C 1s; (**e**) N 1s; (**f**) S 2p.

The elemental composition and valence states of Zn–Co–Fe–S@N–C were analyzed using XPS. Figure 5a shows the XPS signal of Zn 2p, the characteristic peak at 1021.78 eV corresponds to the Zn 2p_3/2_ orbital of Zn^2+^ and the characteristic peak at 1044.82 eV corresponds to the Zn 2p_1/2_ orbital of Zn^2+^. The XPS pattern of Co 2p is shown in Figure 5b, the double peaks of the 2p_3/2_ and 2p_1/2_ spin orbitals of Co^3+^ are located at 779.89 and 794.58 eV, and the 2p_3/2_ and 2p_1/2_ spin–orbit double peaks of Co^2+^ are located at 781.5 and 797.05 eV, respectively. Figure 5c shows Fe 2p XPS peaks, where the characteristic peaks at 709.06 and 722.42 eV correspond to the 2p_3/2_ and 2p_1/2_ orbitals of Fe^2+^, and the characteristic peaks at 711.97 and 725.05 eV correspond to the 2p_3/2_ and 2p_1/2_ orbitals of Fe^3+^. The XPS spectra of C 1s are shown in Figure 5d and the characteristic peaks at 284.8, 286.12 and 288.56 eV correspond to sp^2^-hydrogenated carbon, C–N bonds and O–C=O bonds, respectively. The presence of C-N bonds further confirms the doping of nitrogen in the carbon layer. The presence of O–C=O bonds is mainly due to surface adsorption. As shown in Figure 5e, the high-resolution XPS spectra of N 1s show two peaks at 398.78 and 400.56 eV, which are consistent with the release of pyridine N and pyrrole N from the N-doped carbon material. N doping can improve the electronic conductivity of the carbon layer. Meanwhile, it can also provide more active sites for Li^+^ insertion by generating more defects, which is advantageous for enhancing the electrochemical properties of the electrode. Figure 5f shows the XPS spectrum of S 2p signals, which shows two peaks at 161.51 and 162.69 eV assigned to the S 2p_3/2_ and S 2p_1/2_ orbitals of the M-S bond, indicating the success of sulfidation during the high-temperature calcination.

**Figure 6:**
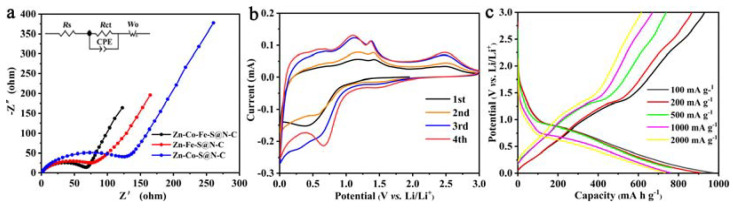
(**a**) Nyquist curves of Zn–Co–Fe–S@N–C, Zn–Co–S@N–C and Zn–Fe–S@N–C; (**b**) the first four CV curves of the Zn–Co–Fe–S@N–C electrode at a scan rate of 0.1 mV s^−1^; (**c**) charge–discharge curves of Zn–Co–Fe–S@N–C at different current densities.

To investigate the differences in the lithium storage processes kinetics of Zn–Co–Fe–S@N–C, Zn–Co–S@N–C and Zn–Fe–S@N–C, EIS measurements were performed on the electrodes, as shown in Figure 6a. Among the illustrations in the figure is a diagram of the equivalent circuit model. The radius of the high-frequency region of Zn–Co–Fe–S@N–C is lower than that of both Zn–Co–S@N–C and Zn–Fe–S@N–C. By fitting the equivalent circuit diagram, the equivalent resistance of Zn–Co–Fe–S@N–C was calculated to be about 67 Ω, and the equivalent resistances of Zn–Co–S@N–C and Zn–Fe–S@N–C were about 75 Ω and 126 Ω, respectively. The excellent electrochemical performance is mainly attributed to the core–shell structure of the nitrogen-doped carbon coating and the synergistic effect of multiple metals, which not only provide sufficient surface-active sites but also shorten the diffusion distance and accelerate the migration rate of Li^+^ ions.

Figure 6b shows the first four CV curves of the Zn–Co–Fe–S@N–C electrode at a scan rate of 0.1 mV s^−1^. During the first cathodic scan, the peak around 0.5 V is probably attributed to the first lithium-ion insertion and the presence of a solid electrolyte interphase (SEI) layer. And on the first anodic scan, the anodic peaks around 1.1, 1.4 and 2.5 V are related to the conversion reaction (Zn→ZnS, Fe→FeS_2_ and Co→CoS_2_) [25,33,34]. A more similar CV curve was shown in the subsequent cycles, indicating good reversibility of the Zn–Co–Fe–S@N–C electrode.

Figure 6c displays the typical charge–discharge curves of Zn–Co–Fe–S@N–C at different current densities. The specific capacities were 971.60 mA h g^−1^ (the 5th cycle), 907.96 mA h g^−1^ (the 10th cycle), 767.44 mA h g^−1^ (the 15th cycle), 759.72 mA h g^−1^ (the 20th cycle) and 732.47 mA h g^−1^ (the 25th cycle) at current densities of 100, 200, 500, 1000 and 2000 mA g^−1^, respectively. The high specific capacity is maintained even at a higher current density of 2000 mA g^−1^, indicating that the core–shell structure and the synergistic effect between the metals contribute to the electrochemical performance. Meanwhile, the unique core–shell structure of Zn–Co–Fe–S@N–C can buffer the volume expansion and provide more active sites to promote the electrochemical reaction, thus improving the electrochemical performance of the anode materials.

**Figure 7:**
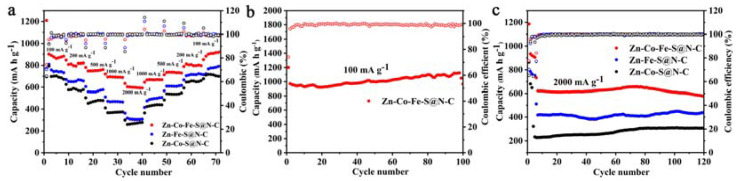
(**a**) Rate performance of Zn–Co–Fe–S@N–C, Zn–Co–S@N–C and Zn–Fe–S@N–C; (**b**) cycling performance of Zn–Co–Fe–S@N–C at a current density of 100 mA g^−1^; (**c**) cycling performance of Zn–Co–Fe–S@N–C, Zn–Co–S@N–C and Zn–Fe–S@N–C at a current density of 2000 mA g^−1^.

The cycling stability of the anodes was evaluated via rate performance analysis (Figure 7a). The specific capacity of Zn–Co–Fe–S@N–C is significantly higher than that of Zn–Co–S@N–C and Zn–Fe–S@N–C. When the current density is 2000 mA g^−1^, the specific capacity of Zn–Co–Fe–S@N–C can reach 666.5 mA h g^−1^. With the decrease in current density, the specific capacity of Zn–Co–Fe–S@N–C also increases gradually. When the current density returns to 100 mA g^−1^, the specific capacity reaches 925.8 mA h g^−1^. The results show that Zn–Co–Fe–S@N–C has good rate performance. In contrast, the specific capacities of Zn–Fe–S@N–C and Zn–Co–S@N–C are 316.7 and 255.1 mA g^−1^, respectively. This also indicates that the multicomposition and core–shell structures have great advantages. A distinctive core–shell structure improves volumetric energy density thanks to the abundant active sites, fast ion mobility, strong electronic conductivity and favorable structural stability; increases the weight fraction of the active material and power density; and extends the cycling performance due to enhanced structural stability [35].

Figure 7b shows the cycling performance and coulombic efficiency of Zn–Co–Fe–S@N–C at a current density of 100 mA g^−1^. The specific capacity gradually increases during the cycling process, mainly because the electrode material is gradually activated as the cycling proceeds. Figure 7c shows the cycling performance and coulombic efficiency of Zn–Co–Fe–S@N–C, Zn–Co–S@N–C and Zn–Fe–S@N–C at 2000 mA g^−1^ current density. The initial discharge capacity of Zn–Co–Fe–S@N–C is 1201.65 mA h g^−1^ and the initial coulomb efficiency is 74.03%. After 120 cycles, the specific capacity is 499.17mA h g^−1^ and the coulomb efficiency reaches 99.71%, showing excellent electrochemical performance. It can be observed from Figure 7c that the specific capacities of Zn–Co–S@N–C and Zn–Fe–S@N–C are significantly lower than those of Zn–Co–Fe–S@N–C. Under the current density of 2000 mA g^−1^, after 120 cycles, the specific capacities are only 221.05 and 320.2 mA h g^−1^, respectively. This illustrates the advantages of multi metals synergies. Among them, Zn–Co–Fe–S@N–C, Zn–Co–S@N–C and Zn–Fe–S@N–C all show good cyclic stability, indicating that the core–shell has more advantages in terms of stability, which is mainly related to the effective mitigation of volume changes by the core–shell structure.

## 3. Experimental Section

### 3.1. Chemicals

All purchased chemicals were directly used without further treatment. Co(NO_3_)_2_·6H_2_O, Zn(NO_3_)_2_·6H_2_O, FeSO_4_·7H_2_O, anhydrous ethanol and anhydrous methanol were purchased from Sinopharm Chemical Reagent Co. Dimethyl imidazole and sublimated sulfur were purchased from Aladdin Corporation. Dopamine hydrochloride and Trizma base were purchased from Sigma–Aldrich Corporation (Darmstadt, Germany). All water used in the experiments involved in this work was ultrapure water.

### 3.2. Preparation of Materials

Synthesis of ZnCoFe–ZIF: 1 mmol Co(NO_3_)_2_·6H_2_O and 10 mmol Zn(NO_3_)_2_·6H_2_O were dissolved in 100 mL methanol and stirred for 30 min. Then, 0.3 mmol FeSO_4_·7H_2_O was added and stirred for 30 min quickly. An amount of 40 mmol of 2–methylimidazole was added to 100 mL methanol, stirred for 30 min, poured into the former solution and stirred at room temperature for 12 h. The resulting precipitate was centrifuged, washed several times with methanol and dried in a vacuum at 60 °C for 12 h.

Synthesis of ZnCoFe–ZIF@PDA: 1 mmol of trimethylol aminomethane (Tris) was added to 100 mL of anhydrous ethanol under ultrasonic conditions. Then, 80 mg ZnCoFe–ZIF precursor was added under ultrasonic treatment for 30 min. An amount of 20 mg of dopamine hydrochloride was added into the mixture and stired for 3.5 h, followed by centrifugation and washing with anhydrous ethanol, and then dried at 60 °C for 12 h.

Synthesis of Zn–Co–Fe–S@N–C: The synthesized ZnCoFe–ZIF@PDA and sulfur powder were carbonized at 600 °C for 2 h with a heating rate of 2 °C min^−1^ under Ar. The Zn–Co–S@N–C and Zn–Fe–S@N–C polyhedrons were also prepared under the same conditions without adding FeSO_4_·7H_2_O or Co(NO_3_)_2_·6H_2_O.

### 3.3. Materials Characterization

The crystal phase of the chemical products was characterized via powder X–ray diffraction (XRD) using a Bruker D8–ADVANCE (40 mA, 40 KV) with Cu Kα (λ = 0.15406 nm) radiation. All samples were observed morphologically using field-emission electron microscopy (FESEM; Hitachi S–4800,Tokyo, Japan). HRTEM images were analyzed withJEM–2100 (JEOL, Tokyo, Japan). X-ray photoelectron spectroscopy (XPS) measurements were collected on an X-ray photoelectron spectrometer (ESCALABXi^+^, Thermo Fisher, Waltham, MA, USA).

### 3.4. Cells Fabrication and Electrochemical Measurements

The prepared samples, Super P and sodium carboxymethylcellulose (CMC) were ground for 30 min at a feeding ratio of 7:2:1. Then, secondary water was added to the mixture under ball milling for 4 h. The copper foil was then coated with a uniform slurry. The coated copper foil was dried in a vacuum-drying oven for 12 h, and then cut into discs with a radius of 6 mm and a loading of about 1.0 mg cm^−2^. The coin cells were assembled in the glove box with lithium foil as the counter electrode, with Celgard 2400 as the separator and 40 μL of 1.0 mol L^−1^ LiPF_6_ (EC:DMC:EMC = 1:1:1) as the electrolyte. The cycle and rate performance of the battery were tested with a voltage range of 0.01~3.0 V. The cyclic voltammetry (CV) curves and impedance spectroscopy measurements were obtained at an electrochemical workstation between 0.01 and 3 V at a scan rate of 0.1 mV s^−1^, and the electrochemical impedance spectroscopy (EIS) tests were performed between around 0.1 and 10,000 Hz.

## 4. Conclusions

In summary, we have prepared Zn–Co–Fe–S@N–C composites through a simple self-templating method as high-performance anode materials for LIBs. Zn–Co–Fe–S@N–C exhibited a high specific capacity and excellent cycling stability. A high reversible capacity of 996.61 mA h g^−1^ was still retained at a current density of 100 mA g^−1^ after 100 cycles. Even at a high current density of 2000 mA g^−1^, the specific capacity of the trimetallic core–shell Zn–Co–Fe–S@N–C polyhedron could still reach 497.72 mA h g^−1^ after 120 cycles. As a result, the rate performance and cycling performance of the Zn–Co–Fe–S@N–C electrodes are superior. Among them, the core–shell structure can provide enough void space to accommodate the volume change during Li^+^ insertion/deinsertion, accelerate the electrolyte transport and shorten the ion/electron diffusion path. The nitrogen-doped carbon shell not only enhances the electrical conductivity of the material, but also improves its structural stability.

## Data Availability

The data that support the findings of this study are available from the corresponding author upon reasonable request.

## References

[1] Cao B., Liu H., Zhang X., Zhang P., Zhu Q., Du H., Wang L., Zhang R., Xu B. (2021). MOF-derived ZnS nanodots/Ti_3_C_2_T*_x_* MXene hybrids boosting superior lithium storage performance. Nano-Micro Lett..

[2] Cai P., Zou K., Zou G., Hou H., Ji X. (2020). Quinone/ester-based oxygen functional group-incorporated full carbon Li-ion capacitor for enhanced performance. Nanoscale.

[3] Zhong Y., Xia X., Shi F., Zhan J., Tu J., Fan H.J. (2016). Transition metal carbides and nitrides in energy storage and conversion. Adv. Sci..

[4] Dong W., Ye B., Cai M., Bai Y., Xie M., Sun X., Lv Z., Huang F. (2023). Superwettable high-voltage LiCoO_2_ for low-temperature lithium-ion batteries. ACS Energy Lett..

[5] Xu C., Feng K., Yang X., Cheng Y., Zhao X., Yang L., Yin S. (2023). In-situ construction of metallic oxide (VNbO_5_) on VNbCT*_x_* MXene for enhanced Li-ion batteries performance. J. Energy Storage.

[6] Yang C., Wang X., Ren Y., Gu S., Wang Q., Li H., Yue K., Gao T., Zhou G. (2023). NiFe_2_V_2_O_8_@N-doped carbon yolk-double shell spheres for efficient lithium storage. Chem. Eng. J..

[7] Ren Y., Li X., Wang Y., Gong Q., Gu S., Gao T., Sun X., Zhou G. (2022). Self-template formation of porous yolk-shell structure Mo-doped NiCo_2_O_4_ toward enhanced lithium storage performance as anode material. J. Mater. Sci. Technol..

[8] Gao L., Liu Y., Hao Y., Zheng Z., Kong S., Zhang L., Yang X. (2022). Self-supported selenium doped Fe_2_P hierarchical microsphere arrays enabling high performance sodium ion batteries. J. Alloys Compd..

[9] Xia Y., Yang T., Wang Z., Mao T., Hong Z., Han J., Peng D.-L., Yue G. (2023). Van der Waals Forces between S and P Ions at the CoP-C@MoS_2_/C Heterointerface with Enhanced Lithium/Sodium Storage. Adv. Funct. Mater..

[10] Li H., He Y., Dai Y., Ren Y., Gao T., Zhou G. (2022). Bimetallic SnS_2_/NiS_2_@S-rGO nanocomposite with hierarchical flower-like architecture for superior high rate and ultra-stable half/full sodium-ion batteries. Chem. Eng. J..

[11] Zhang Z., Huang Y., Liu X., Chen C., Xu Z., Liu P. (2020). Zeolitic imidazolate frameworks derived ZnS/Co_3_S_4_ composite nanoparticles doping on polyhedral carbon framework for efficient lithium/sodium storage anode materials. Carbon.

[12] Guo Z., Zhang M., Wu F., Su Z., He L., Zhou P., Xu P., Zou R., Wang X., Huang Q. (2021). Honeycomb-structured α-MnS@N-HC nanocomposite fabricated by sol-gel pyrolysis blowing method and its high-performance lithium storage. Mater. Today Energy.

[13] Lin Y., Qiu Z., Li D., Ullah S., Hai Y., Xin H., Liao W., Yang B., Fan H., Xu J. (2018). NiS_2_@CoS_2_ nanocrystals encapsulated in N-doped carbon nanocubes for high performance lithium/sodium ion batteries. Energy Storage Mater..

[14] Chiu J.-M., Chou T.-C., Wong D.P., Lin Y.-R., Shen C.-A., Hy S., Hwang B.-J., Tai Y., Wu H.-L., Chen L.-C. (2018). A synergistic “cascade” effect in copper zinc tin sulfide nanowalls for highly stable and efficient lithium-ion storage. Nano Energy.

[15] Nong Y., Zhang M., Li Q., Pan Q., Huang Y., Wang H., Zheng F., Li Q. (2022). Carbon coated bimetallic sulfides Co_9_S_8_/ZnS heterostructures microrods as advanced anode materials for lithium-ion batteries. J. Taiwan Inst. Chem. Eng..

[16] Jin R., Wang G., Gao S., Kang H., Chen S. (2021). NiS_1.03_@NiMoS_4_ nanocrystals encapsulated into the mesoporous carbon microspheres for high performance lithium ion batteries. J. Electroanal. Chem..

[17] Xiao Y., Hwang J.-Y., Belharouak I., Sun Y.-K. (2017). Superior Li/Na-storage capability of a carbon-free hierarchical CoS*_x_* hollow nanostructure. Nano Energy.

[18] He Z., Guo H., LaCoste J.D., Cook R.A., Hussey B., Zhang X., Gang D.D., Hao J., Chen L., Cooke P. (2021). Directly embedded Ni_3_S_2_/Co_9_S_8_@S-doped carbon nanofiber networks as a free-standing anode for lithium-ion batteries. Sustain. Energy Fuels.

[19] Fang G., Wang Q., Zhou J., Lei Y., Chen Z., Wang Z., Pan A., Liang S. (2019). Metal organic framework-templated synthesis of bimetallic selenides with rich phase boundaries for sodium-ion storage and oxygen evolution reaction. ACS Nano.

[20] Zhang H., Zhao L., Ye L., Li G. (2021). Capacity and cycle performance of lithium ion batteries employing Co*_x_*Zn_1−*x*_S/Co_9_S_8_@N-doped reduced graphene oxide as anode material. Chem. Eng. J..

[21] Li L., Zhao J., Zhao H., Mao J. (2022). Bi_2_Se_0.5_Te_2.5_/S, N-doped reduced graphene oxide as anode materials for high-performance Lithium ion batteries. J. Alloys Compd..

[22] Jiang S., Suo H., Zheng X., Zhang T., Lei Y., Wang Y., Lai W., Wang G. (2022). Lightest metal leads to big change: Lithium-mediated metal oxides for oxygen evolution reaction. Adv. Energy Mater..

[23] Wang X., Zhang S., Shan Y., Chen L., Gao G., Zhu X., Cao B., He X. (2021). In situ heterogeneous interface construction boosting fast ion/electron transfer for high-performances lithium/potassium storage. Energy Storage Mater..

[24] Wang L., Wan J., Zhao Y., Yang N., Wang D. (2019). Hollow multi-shelled structures of Co_3_O_4_ dodecahedron with unique crystal orientation for enhanced photocatalytic CO_2_ reduction. J. Am. Chem. Soc..

[25] Fang Y., Luan D., Chen Y., Gao S., Lou X.W. (2020). Synthesis of copper-substituted CoS_2_@Cu*_x_*S double-shelled nanoboxes by sequential ion exchange for efficient sodium storage. Angew. Chem. Int. Ed..

[26] Zhang J., Wan J., Wang J., Ren H., Yu R., Gu L., Liu Y., Feng S., Wang D. (2019). Hollow Multi-Shelled Structure with Metal–Organic-Framework-Derived Coatings for Enhanced Lithium Storage. Angew. Chem. Int. Ed..

[27] Gui Q., Feng Y., Chen B., Gu F., Chen L., Meng S., Xu M., Xia M., Zhang C., Yang J. (2021). Extrinsic-structured bimetallic-phase ternary metal phosphorus trisulfides coupled with N-doped graphitized carbon for superior electrochemical lithium storage. Adv. Energy Mater..

[28] Peng J., Li Y., Chen Z., Liang G., Hu S., Zhou T., Zheng F., Pan Q., Wang H., Li Q. (2021). Phase compatible NiFe_2_O_4_ coating tunes oxygen redox in Li-rich layered oxide. ACS Nano.

[29] Aslam M.K., Shah S.S.A., Li S., Chen C. (2018). Kinetically controlled synthesis of MOF nanostructures: Single-holed hollow core–shell ZnCoS@Co_9_S_8_/NC for ultra-high performance lithium-ion batteries. J. Mater. Chem. A.

[30] Wei Y., Wang Z., Wang J., Bai W., Zhang Y., Liu B. (2023). Designing of trimetallic-phase ternary metal sulfides coupled with N/S doped carbon protector for superior and safe Li/Na storage. J. Colloid Interface Sci..

[31] Cao L., Li Z., Su K., Zhang M., Cheng B. (2021). Rational design of hollow oxygen deficiency-enriched NiFe_2_O_4_@N/rGO as bifunctional electrocatalysts for overall water splitting. J. Energy Chem..

[32] Wang D., Zhou C., Cao B., Xu Y., Zhang D., Li A., Zhou J., Ma Z., Chen X., Song H. (2020). One-step synthesis of spherical Si/C composites with onion-like buffer structure as high-performance anodes for lithium-ion batteries. Energy Storage Mater..

[33] Yang P., Yuan Y., Zhang D., Yang Q., Guo S., Cheng J. (2023). Nanocapsule of MnS nanopolyhedron core@CoS nanoparticle/carbon shell@pure carbon shell as anode material for high-performance lithium storage. Molecules.

[34] Wang J., Lin Y., Lv W., Yuan Y., Guo S., Yan W. (2023). Bismuth-antimony alloy nanoparticles embedded in 3D hierarchical porous carbon skeleton film for superior sodium storage. Molecules.

[35] Zhu S., Yuan Y., Du P., Zhu M., Chen Y., Guo S. (2023). α-MnS nanoparticles in-situ anchored in 3D macroporous honeycomb carbon as high-performance anode for Li-ion batteries. Appl. Surf. Sci..

